# SARS-CoV-2 infection in fully vaccinated patients with multiple myeloma

**DOI:** 10.1038/s41408-021-00597-y

**Published:** 2021-12-14

**Authors:** Nicola Sgherza, Paola Curci, Rita Rizzi, Immacolata Attolico, Daniela Loconsole, Anna Mestice, Maria Chironna, Pellegrino Musto

**Affiliations:** 1 Hematology and Bone Marrow Transplantation Unit, AOUC Policlinico, Bari, Italy; 2 Department of Emergency and Organ Transplantation, “Aldo Moro” University School of Medicine, Bari, Italy; 3 Department of Biomedical Sciences and Human Oncology, “Aldo Moro” University School of Medicine, Bari, Italy

**Keywords:** Myeloma, Infectious diseases

“Coronavirus Disease 2019” (COVID-19) due to SARS-CoV-2 infection is characterized by a poorer outcome in patients with hematologic malignancies [1, 2]. Specifically, several papers have reported more frequent and severe COVID19, as well as higher fatality rates, in patients with multiple myeloma (MM), particularly in those older than sixty, with high risk, active/progressive disease, and/or renal failure [3–5]. On this basis, the International Myeloma Society recommends vaccination for SARS-CoV-2 for all patients with MM (https://cms.cws.net/content/beta.myelomasociety.org/files/PM%20COVID%20vaccination%20in%20MM%20guidelines%20The%20Final.pdf).

MM patients, however, show an immune dysregulation attributable to the disease itself or to anti-tumor treatments. For this reason, they were excluded from initial anti-SARS-CoV-2 vaccine clinical trials. As a consequence, efficacy, durability, and safety of COVID-19 vaccines in these immunocompromised subjects are yet to be fully established [6]. Indeed, low antibody responses have been reported among elderly MM patients who had received the first dose of the BNT162b2 COVID-19 mRNA vaccine [7]. These data were supported by another study demonstrating a suboptimal response after vaccination, especially in subjects on treatment with anti-CD38-based regimens [8]. A recent study confirmed that fully vaccination with either the BNT162b2 mRNA or the AZD1222 viral vector vaccine leads to a less intense humoral response, as reflected by a lower production of neutralizing antibodies against SARS-CoV-2, among patients with MM or smoldering MM (SMM) compared with healthy controls [9]. Importantly, active treatment with either anti-CD38 or anti-BCMA monoclonal antibodies, lymphopenia and immunoparesis at the time of vaccination were independent prognostic factors for suboptimal antibody response [9].

The possibility of SARS-CoV-2 infection in fully vaccinated patients is a relevant clinical issue in the general population [10] and in immunocompromised patients, particularly after solid organ transplantation [11, 12]. Some patients with MM developing COVID19 after anti-SARS-CoV-2 vaccination have been recently reported in a multicenter study including many other hematologic malignancies [13]. However, no detailed data are currently available about specific clinical and laboratory characteristics of these patients.

We describe here five patients affected by MM or SMM, who resulted positive for SARS-CoV-2 infection by real-time reverse-transcriptase PCR on nasopharyngeal swabs from June 2021 to September 2021, despite they had received two doses of BNT162b2 COVID-19 mRNA vaccine (Table 1). These patients belonged to a cohort of 260 MM patients (including subjects with SMM) currently followed at our Institution and fully vaccinated with BNT162b2 COVID-19 mRNA vaccine between March and June 2021. Written informed consent was obtained from each patient within the context of the ClinicalTrials.gov Identifier NCT04492371.

**Table 1:** Clinical and laboratory characteristics of vaccinated MM patients with SARS-CoV-2 infection.

Pt	Age (yrs)	Sex	Comorbidities	Tipe of MM	Hematologic therapies at the time of infection	Previous therapies for MM	Immunoparesis^a^	Lympho penia (<1000/ mm^3^)	Date of diagnosis of SARS-CoV-2 infection	Date of first/second dose of BNT162b2 mRNA vaccine	Days from fully vaccination to SARS-CoV-2 infection	COVID-19 Symptoms	SARS-CoV-2 Variant of Concern	Anti-spike IgG^b^ before infection/days from fully vaccination	Anti-spike IgG^b^ post infection/days from fully vaccination	Anti SARS-CoV-2^c^ IgM/IgG days from infection
1	76	F	Hypertension Severe obesity Chronic obstructive bronchopathy Chronic renal failure	SMM, IgA λ	No	No	No	No	03/06/2021	21/04/2021 12/05/2021	21	Diarrhea	Alpha (lineage B.1.1.7)	N.A.	2812/184	IgM−/IgG− 162
2	71	F	Diabetes mellitus Hypothyroidism	MM, IgG k	Radiotherapy	No	Yes	Yes	09/08/2021	22/04/2021 18/05/2021	83	Dry cough	Delta (lineage B.1.617.2)	N.A.	495/178	IgM−/IgG− 95
3	56	M	Hypertension Glomerulonephritis with C3-deposits Kidney-transplant recipient	SMM, IgG λ	Cyclosporin	No	No	No	24/08/2021	27/03/2021 17/04/2021	129	Fever Pneumonia	Delta (lineage B.1.617.2)	N.A	592/205	IgM−/IgG+ 76
4	70	M	Hypertension	MM, IgG k	DRd	VAD, sASCT, VTD	No	No	06/09/2021	06/05/2021 12/06/2021	86	No	Delta (lineage B.1.617.2)	N.A.	682/149	IgM+/IgG− 63
5	54	F	Hypothyroidism	MM, IgG k	Lenalidomide	VTD, dASCT	Yes	Yes	10/09/2021	01/04/2021 23/04/2021	140	Fever	Delta (lineage B.1.617.2)	828/30	26710/199	IgM−/IgG+ 59

*dASCT*: double autologous stem cell transplantation; *DRd*: daratumumab, lenalidomide, dexamethasone; *MM*: multiple myeloma; *N.A*.: not available; *sASCT*: single autologous stem cell transplantation; *Pt* patient; *SMM*: smoldering multiple myeloma; *yrs*: years; *VAD*: vincristine, doxorubicin, dexamethasone; *VTD*: bortezomib, thalidomide, dexamethasone.

^a^Reduced levels of at least one not involved immunoglobulin.

^b^Chemiluminescent microparticle immunoassay (CMIA) technology: results were reported as arbitrary units (AU), with a positivity cut-off level of ≥50 AU/ml.

^c^CMIA qualitative test: negative (−) or positive (+).

Case 1: 76-year-old white woman, with IgA λ SMM (diagnosis January 2015), hypertension, severe obesity, chronic obstructive bronchopathy, and chronic renal failure (III/IV K-DOQI). SARS-CoV-2 infection manifested with diarrhea for a few days, in June 2021 (21 days after the second dose of vaccine).

Case 2: 71-year-old white woman, with IgG k MM, stage II ISS-R (diagnosis June 2021), diabetes mellitus, hypothyroidism. She underwent radiotherapy on the right iliac wing on July 2021; in August 2021, just the day before starting the first cycle of planned immune-chemotherapy with daratumumab, lenalidomide, and dexamethasone (DRd), the patient presented dry cough and SARS-CoV-2 infection was diagnosed after 83 days from the second dose of vaccine. The duration of the symptoms was about ten days.

Case 3: 56-year-old black man, kidney-transplant recipient (under cyclosporine treatment), with IgG λ SMM and hypertension. SARS-CoV-2 infection was diagnosed on August 2021, by a nasopharyngeal swab performed after returning from a trip to Africa and 129 days from the second dose of vaccine. After few days of well-being, he was hospitalized for fever and pneumonia and treated with antibiotics and steroids; oxygen therapy was not necessary. He was discharged after a 10-day hospitalization and complete resolution of the clinical picture, without sequelae.

Case 4: 70-year-old white man, with hypertension and relapsed MM IgG k, stage II ISS (diagnosis of SMM in October 2000), now receiving DRd (19 cycles, until August 2021). Previous therapies, started in 2003 for progressive disease, included: vincristine, doxorubicine and dexamethasone (VAD), single autologous stem cell transplantation (ASCT), and bortezomib, thalidomide, and dexamethasone (VTD). He was asymptomatic when and after SARS-CoV-2 infection was confirmed in September 2021, through a nasopharyngeal swab planned, according to our Institution’s internal policy, before the 20th DRd cycle and after 86 days from the second dose of vaccine.

Case 5: 54-year-old white woman, with hypothyroidism and IgG k MM, stage I ISS (diagnosis December 2018), now receiving lenalidomide maintenance (after VTD induction, and double ASCT). SARS-CoV-2 infection manifested with fever for a few days, in September 2021 (after 140 days from the second dose of vaccine). The patient participated to a clinical study on serological response to anti-SARS-CoV-2 vaccination in patients with a prior history of either autologous or allogeneic hematopoietic stem cell transplantation [14]. In this patient, an apparently appropriate serological response was found one month after the second dose of BNT162b2 COVID-19 mRNA vaccine (see below).

An in-depth analysis of viral genotype was performed in all patients above described (Table 1). Patient n.1 showed an S-gene target failure (SGTF) at real-time PCR, which could be considered a robust proxy of Alpha SARS-CoV-2 Variant of Concern (VOC lineage B.1.1.7). To confirm the presence of B.1.1.7 VOC, the sample was also screened for the presence of notable spike protein mutations using a commercial multiplex real-time PCR kit. Samples collected from patients 2–5 were SGTF-negative, suggesting the presence of a SARS-CoV-2 variant other than B.1.1.7. The same commercial multiplex real-time PCR kit confirmed the presence of Delta SARS-CoV-2 Variant of Concern (VOC lineage B.1.617.2) in these patients. Thus, viral genotype in our fully vaccinated MM patients followed the current epidemiological diffusion in Italy, with a clear predominance of Delta variant. Infection occurred after a median of 86 days, (range 21–140 days) from the second dose of vaccine.

Quantitative determination of anti-spike IgG antibodies (evaluating humoral response to vaccination), as well as qualitative anti-SARS-CoV-2 tests, specifically evaluating exposure to the virus (IgG and IgM), were performed using a chemiluminescent microparticle immunoassay technology. Serum samples for anti-spike IgG antibodies detection collected after vaccination and before the evidence of SARS-CoV-2 infection were available in one patient (case 5) enrolled in a clinical study, where this type of analysis had been planned [14]. In this patient, detection of anti-spike IgG antibodies revealed a serum titer of 828 AU/ml four weeks after the second dose of BNT162b2 COVID-19 mRNA vaccine (Table 1), though it was much lower than the median value (7.132 AU/ml) detected in healthy controls enrolled in the study. Interestingly, serum levels of anti-spike IgG antibodies significantly increased in this patient to 26.710 AU/ml two months after SARS-CoV-2 infection (Table 1). This finding would seem to reinforce the concept that a further immunologic stimulus deriving from a contact with the virus (but it could be also the case of a “third dose”), is probably able to (re)generate a robust, new serological response in fully vaccinated patients. Unfortunately, serological data before SARS-CoV-2 infection regarding the other four patients were not available to confirm this hypothesis. Anti-spike IgG antibodies after SARS-CoV-2 infection were instead available and detected in all patients, showing variable titers (Table 1).

Regarding specific anti-SARS-CoV-2 antibodies, IgM were positive in one patient 63 days after infection, while IgG were detected (with negative IgM) in two patients after 76 and 59 days, respectively. Both IgG and IgM were negative in the remaining two patients after a longer period of time (95 and 162 days, respectively) (Table 1).

Two patients also had immunoparesis and lymphopenia (considered predictive factors for suboptimal antibody response following vaccination) before infection (Table 1), but only the patient on treatment with anti-CD38 monoclonal antibody daratumumab showed a very low count (1%) of CD19 + B-lymphocytes by flow cytometry in peripheral blood. One patient also was a kidney transplant recipient, a condition at higher risk of severe COVID-19, even though fully vaccinated [11, 12]. Other comorbidities were also frequent (Table 1). Notwithstanding, four patients had very few or no symptoms, did not require hospitalization or specific anti-viral treatment for COVID-19 and rapidly recovered; only one patient, the kidney-transplant recipient, was hospitalized for a few days in an ordinary care unit and treated with antibiotics and steroids for pneumonia, with a rapid resolution of the clinical picture.

Our data refer to only five patients and are certainly very preliminary. However, taking into account the fatality rates of 26–58% reported for non-vaccinated MM patients with COVID-19 [1–3], our findings support the hypothesis of a “protective” effect of vaccination against the severity of COVID-19 (particularly in preventing death and hospitalization in intensive care unit) also in a group of patients with MM and SMM, some of whom were particularly at risk.

Obviously, several limitations are present in our analysis. First, the small number of patients described; further data, from a higher number of subjects enrolled preferably within multicenter studies, are needed to achieve greater generalizability of our findings. Second, serum samples for anti-spike IgG detection after vaccination were available only for one patient; it would have been interesting to evaluate the antibody titer of all patients before SARS-CoV-2 infection. Third, although in our center a nasopharyngeal swab for SARS-CoV-2 is routinely performed within 48/72 h before every infusion treatment, infection might be misclassified in asymptomatic, vaccinated MM patients unaware of being infected.

In conclusion, the clinical outcome of COVID-19 may be favorable after vaccination in MM patients, even in the presence of negative prognostic factors. However, vaccinated MM patients remain at risk of acquiring SARS-CoV-2, so their continuous monitoring and testing is advisable. Furthermore, they should continue to practice strict ongoing protective measures, as well as prioritize vaccination for family members and caregivers, particularly in light of the worldwide worrisome spread of SARS-CoV-2 variants.
